# Feasibility of introducing a smartphone navigation application into the care of breast cancer patients (The FIONA Study)

**DOI:** 10.1007/s10549-023-06918-y

**Published:** 2023-04-27

**Authors:** Steven J. Isakoff, Maya R. Said, Agnes H. Kwak, Eva Glieberman, Emily A. O’Rourke, Amanda Stroiney, Laura M. Spring, Beverly Moy, Aditya Bardia, Nora Horick, Jeffrey M. Peppercorn

**Affiliations:** 1 Massachusetts General Hospital Cancer Center, 55 Fruit Street, Boston, MA 02114 USA; 2 Outcomes4Me Inc, One Beacon St, 15th Floor, Boston, MA 02108 USA

**Keywords:** Mobile technology, Patient education, Symptom reporting, Breast cancer, App, Patient engagement

## Abstract

**Purpose:**

Patients with breast cancer (BC) face complex medical information and decisions. The Outcomes4Me mobile app provides evidence-based BC education, symptom management tracking and clinical trial matching. This study sought to evaluate the feasibility of introducing this app into routine BC care.

**Methods:**

In this pilot study among BC patients undergoing therapy at an academic cancer center, patients were followed for 12 weeks with survey administration and electronic health record (EHR) abstraction at baseline and completion. Feasibility was defined as 40% of patients engaging with the app 3 or more times during the study. Additional endpoints included app usability (system usability scale), patient care experience, symptom evaluation, and clinical trial matching.

**Results:**

The study enrolled 107 patients from 6/01/2020 to 3/31/2021. Utilization of the app was deemed feasible with 60% of patients engaging with the app at least 3 times. SUS score of 70 indicated above average usability. New diagnosis and higher education level was associated with greater app engagement, with usability similar across all age groups. 41% of patients found the app helped track symptoms. Cognitive and sexual symptoms were infrequently reported, but were more frequently captured in the app than in the EHR. After using the app, 33% of patients reported increased interest in clinical trial enrollment.

**Conclusion:**

Introducing the Outcomes4Me patient navigation app into routine BC care is feasible and may improve the patient experience. These results support further evaluation of this mobile technology platform to improve BC education, symptom management, and decision making.

**Clinical trial registry:**

Clinicaltrials.gov registration #: NCT04262518

**Supplementary Information:**

The online version contains supplementary material available at 10.1007/s10549-023-06918-y.

## Introduction

In all phases of cancer care, from new diagnosis, to survivorship, to living with metastatic disease, patients with breast cancer face unique health challenges, a large volume of information, and complex health care decisions. Multiple studies demonstrate the need and desire for improved understanding of diagnosis, prognosis, and treatment options among patients with breast cancer [1–5]. Studies also suggest that patients who are more engaged in their care and who use a digital device for symptom self-reporting experience better outcomes. [6–10] The landmark study by Basch and colleagues demonstrated that patient use of electronic tablets to self-report symptoms which are then provided to the care team resulted in improved quality of life and overall survival [9, 10]. While the value of patient education, engagement, and shared decision-making to promote self-efficacy and individualized cancer care is well recognized, there is no tool or technology that is routinely used to achieve these ends [11, 12]. In this context, there is an unmet need and an opportunity to develop a tool to help educate patients about their disease, symptoms, and care options, and help them navigate the complex web of medical decisions to get individualized care that matches their specific case and preferences.

In this increasingly digital age, the incorporation of technology in healthcare has increased the potential for information dissemination. While there are many technological resources available to patients with breast cancer, there are few comprehensive, patient-centered tools available to educate patients about their disease, symptoms, and care options, while providing personalized support and care management. The Outcomes4Me app was designed in collaboration with breast cancer patient advocates and clinicians to provide a convenient and accessible resource that could help patients understand their diagnosis, navigate treatment options, and track and manage symptoms.

In this single-arm pilot study, we sought to assess the feasibility of incorporating the Outcomes4Me smart phone navigation application into the standard of care experience of breast cancer patients across the cancer care continuum. We also sought to evaluate the patient experience with the app, identify preferences for education, symptom tracking and other features, and explore the impact of the app on perceptions of care management, patient-provider interactions, and understanding of disease and treatment options.

## Methods

### Study design and patient population

The Feasibility of Introducing the Outcomes4Me Smartphone Navigation App (FIONA) trial is single-arm prospective study evaluating the feasibility of introducing a breast cancer education and navigation app into routine breast cancer care. The study was conducted among patients presenting for breast cancer care at the Massachusetts General Hospital Cancer Center or community-based satellite clinics. Eligible patients included English-speaking adults with invasive breast cancer stage I-IV presenting for a new diagnosis or follow-up visit who had access to an Apple or Android smartphone. Eligibility required that the patient was planning to receiving some form of active treatment for breast cancer (including surgery, radiation therapy, chemotherapy, endocrine therapy, or targeted therapy) within 4 weeks of study entry and was expected to continue follow-up within the cancer center. Eligible patients were identified by screening clinic lists and patients were recruited in-person or by telephone with electronic informed consent.

After signing consent, participants were asked to download the Outcomes4Me app onto their smartphone with assistance from the research coordinator. Study usernames and emails were provided to each participant to create an anonymous app account. Participants were surveyed at baseline and at completion of the study at 12 weeks. In addition, participants received weekly app notifications to report any symptoms. The study was approved by the Dana-Farber/Harvard Cancer Center Institutional Review Board.

### Navigation app development and description of app features

The app was developed by Outcomes4Me, a for-profit digital health company based in Boston, Massachusetts. The app involves patient engagement in multiple domains including breast cancer education, treatment guidelines, potential side effects and management, symptom tracking, medication tracking, clinical trial matching, and note taking. The app also provides a newsfeed about breast research in the media.

Treatment and side effect information, symptom tracking and clinical trial matching provide evidence-based, personalized information for patients. Information about medication side effects and treatment options are adapted from Wolters Kluwer Health and NCCN Clinical Practice Guidelines in Oncology (NCCN Guidelines®) for Breast Cancer under an approved use agreement. Information about breast cancer and treatment is tailored to disease characteristics entered by patients, such as hormone receptor status, HER2 status, and stage.

The app symptom tracking feature enables patients to enter symptom type, severity, and frequency using PRO-CTCAE criteria. Clinical trial matching is based on patient entered disease characteristics using an automated algorithm that presents a list of trials generated from clinicaltrials.gov.

### Survey development and domains

A baseline and follow-up survey were developed to evaluate the following domains: demographics, breast cancer history, emotional distress, health care information practices and preferences, and understanding of disease and treatment plan. The surveys were developed by a multidisciplinary group of investigators with expertise in breast oncology, survey design, biostatistics, and health technology. Distress and emotional concerns were evaluated with the National Comprehensive Cancer Network Distress Thermometer and study specific questions about anxiety and satisfaction with care [13]. The patient’s relationship with their oncologist was evaluated using the Patient-Doctor Relationship Questionnaire (PDRQ9) [14]. A similar follow-up survey was administered after 12 weeks, with additional questions to evaluate patient experience with the app including the System Usability Scale (SUS), a validated usability measure ranging from 0 to 100 with higher scores indicating greater usability, and the Net Promotor Score (NPS), a measure of overall satisfaction calculated by subtracting the percentage of “detractor” participants who are unlikely to recommend the app to a friend (0–6 on 10-point scale) from the percentage who are “promoters” and highly likely to recommend the app (9–10) [15–17]. In addition, study specific questions evaluated the participants’ experience with the app and suggestions for improvement.

### Statistical analysis

The primary endpoint was feasibility of using the application in a clinical setting, with feasibility demonstrated if at least 40% of all enrolled participants engaged with the app at least three times during the 12-week study period. Engagement was defined as login and interaction with at least one app feature. Login to complete the baseline and follow-up survey was not included. Secondary endpoints included the average SUS and the NPS. The average SUS score in the literature is 68, and the study set 70 or above as a measure of usability [18]. The study was initially designed to recruit 125 patients including 20 newly diagnosed, 20 on adjuvant therapy, and 50 with metastatic disease. However, delays in study initiation due to COVID-19 resulted in a modified recruitment of 110 patients.

Descriptive statistics for survey responses including frequencies, proportions, medians, and means were computed. Exploratory analyses evaluated the association between participants’ sociodemographic and clinical characteristics and their level of engagement (≥ 5 versus < 5 interactions during study period) and overall satisfaction (≥ 7 versus < 7, corresponding to NPS “promoter” or “passive” versus “detractor”) with the app using Fisher’s exact test. Additional exploratory analyses assessed the association between participant characteristics and SUS using the Kruskal–Wallis test. A two-sided significance level of 0.05 was used, without adjustment for multiple comparison due to the exploratory nature of the analyses.

## Results

### Patient sample, demographics, and baseline preferences for information

**Fig. 1:**
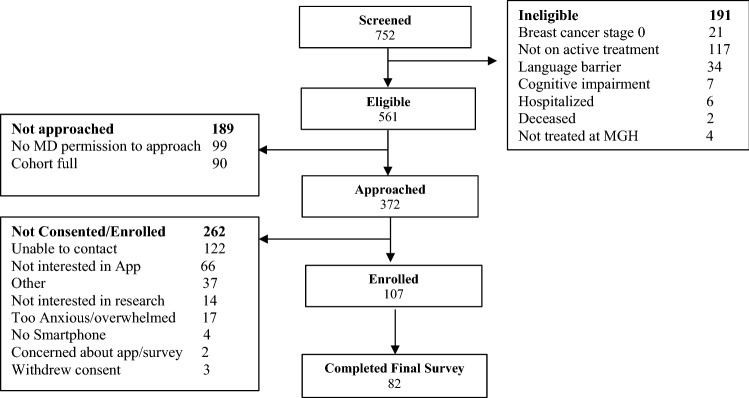
Screening and Enrollment to the FIONA Study

Between June 1st, 2020 and December 31st, 2020, 110 patients were recruited and registered to the study. Three patients withdrew consent prior to downloading the app and completing the baseline survey, resulting in 107 evaluable at baseline, and 82 patients (75%) completed the final survey. Details of patient disposition including screening and recruitment are presented in Fig. 1.

Demographics and clinical characteristics of enrolled participants is shown in Table 1. All 107 were women, with average age 53 (range 27 to 77). Twenty percent were newly diagnosed and 45% were receiving adjuvant treatment, of whom 52% and 19% were receiving chemotherapy, respectively, and 36% had metastatic breast cancer.

**Table 1:** Clinical and demographic characteristics of study participants

		N (%)
Age	18–39	9 (8)
	40–69	88 (82)
	70 +	10 (9)
Race	White	96 (90)
	Black or African American	5 (5)
	Asian or Asian Indian	3 (3)
	Other or prefer not to answer	3 (3)
Education	High school through junior college	21 (20)
	College degree (B.A/B.S)	40 (37)
	Post college education	46 (43)
Breast cancer subtype	Hormone positive/HER2-	72 (67)
	HER2 +	20 (19)
	Triple Negative	15 (14)
Treatment setting and treatment type	Newly diagnosed Chemotherapy Endocrine therapy Adjuvant therapy Chemotherapy Endocrine Metastatic therapy Chemotherapy Endocrine Radiation Surgery	21 (20) 11 (52) 6 (29) 48 (45) 9 (19) 39 (81) 39 (36) 12 (31) 26 (67) 11 (10) 10 (9)
Smartphone health app	Use	99 (93)
	Don’t Use	8 (7)

### Baseline patient preferences on information sources

At baseline, patients were asked which sources of information they relied upon for information about their cancer. Most patients reported relying on their doctors (99%), other people on their cancer care team (82%), cancer organizations (64%), and family and friends (55%). Forty two percent of patients used a general internet search for information, and 25% relied on patient support groups. At baseline, 93% of patients reported that they did not use any healthcare related apps (Table 1) and only 2% of participants listed health as one of the top uses for their mobile device.

### Feasibility and usability

**Fig. 2:**
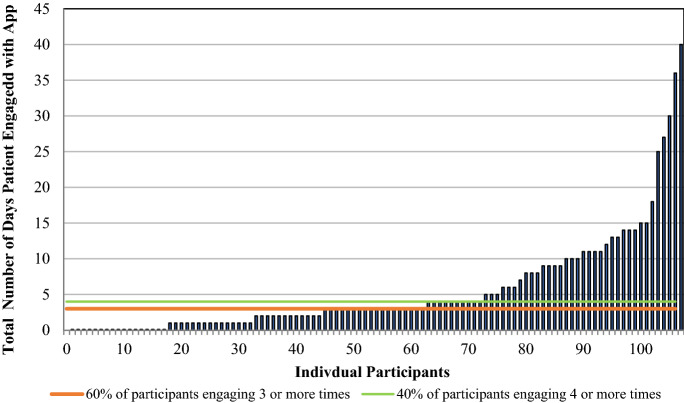
Patient Engagement with Outcomes4Me App. The distribution of individual patient engagement for all 107 patients during the study period is shown. Orange line denotes patients who engaged 3 or more times with the app; green line denotes patients who engaged 4 or more times with the app

Integration of the Outcomes4Me app into the routine care of patients with breast cancer in active treatment was deemed feasible with 60% of participants engaging with the app at least 3 times during the study period, exceeding the prespecified target of 40%. There was wide variation in the level of engagement with the app during the study period among all participants, as shown in Fig. 2. Across all participants and for the subset with metastatic disease, median engagement was 3. Among all newly diagnosed patients the median engagement was 6, and for patients receiving chemotherapy for early-stage disease it was 4. Patients who engaged with the app 3 or more times were more likely to have at least a college degree (94% v. 74%, *p* = 0.02) and more likely to be newly diagnosed (34% v. 13%, *p* = 0.03) than those with less frequent engagement.

The mean usability score, as demonstrated by the SUS was 70 (median 76), exceeding the threshold for “above average” usability of 68 [18]. Among patients receiving chemotherapy for early-stage disease, it was 74, for newly diagnosed patients it was 74, and for patients with metastatic disease it was 66. Among patients between 18 and 39 years old, the usability score was 75, for patients 40–69 it was 70 and for patients older than 70 it was 74.

The overall NPS was − 37, suggesting 37 more detractors (respondents with a score of 0–6 on 10-point scale of willingness to recommend the app to other patients) then promotors (respondents with a score of 9 or 10) out of 100 app user. While the ratio of promotors to detractors of the app increased among more frequent users, the NPS was still negative, at—28 among those who engaged with the app 5 times or more, compared to − 41 among those who were less engaged. Among patients receiving adjuvant chemotherapy the NPS was balanced, with a score of 0 (promotors equal detractors). Tendency to promote the app, based on NPS, seemed to vary with stage with an NPS score of—47 among patients with stage I disease (*n* = 32) 0 among patients with stage II (*n* = 18), and 14 among patients with stage III (*n* = 7). However, among patients with metastatic disease it was -57.

### Symptom reporting and tracking

At least one symptom was reported by 43% (46) of participants, and 40% (43) reported multiple symptoms through the app. The most commonly reported symptoms were: GI issues (30%), sleep disturbance (29%), pain (28%), mood issues (18%), and neurologic issues (17%) (Supplementary Materials Table 1).

Interestingly, several symptoms were reported more frequently by patients in the app than documented by providers in the EHR. Cognitive issues, sexual health problems (decreased libido), and depression were rare (< 10%), but were reported in the app by at least 5% of participants, and virtually never reported in the EHR (Table 2). This trend was even more pronounced when analysis was restricted to the subset of participants with at least one follow-up clinic visit during the study period and at least 5 episodes of engagement with the app. Nausea was more frequently recorded in the EHR than reported in the app. Most differences observed between symptom reporting in the app vs. documentation in the EHR were not statistically significant.

**Table 2:** Most Commonly Reported Symptoms in App and Electronic Health Record (EHR) among Total Study Population and Population Highly Engaged with App

Symptom	Symptoms reported by all participants (*N *= 107)		Symptoms reported by participants with at least 5 engagement episodes (*N* = 35)	
	*N* (%) reporting ever in app	*N* (%) reporting ever in EHR	*N* (%) reporting ever in app	*N* (%) reporting ever in EHR
Attention problems	7 (7%)	1 (1%)	5 (14%)	1 (3%)
Anxiety	13 (12%)	9 (8%)	10 (29%)	5 (14%)
Constipation	10 (9%)	13 (12%)	8 (23%)	3 (9%)
Decreased libido	5 (5%)	0	3 (9%)	0
Depression	8 (7%)	1 (1%)	6 (17%)*	0
Diarrhea	20 (19%)	24 (22%)	14 (40%)	11 (31%)
Fatigue	29 (27%)	44 (41%)	22 (63%)	16 (46%)
Headache	14 (13%)	14 (13%)	11(31%)	4 (11%)
Hot flashes	15 (14%)	17 (16%)	8 (23%)	3 (9%)
Insomnia	15 (14%)	16 (15%)	11 (31%)	8(23%)
Memory Difficulty	7 (7%)	1 (1%)	5 (14%)	0
Nausea	14 (13%)	27 (25%)*	9 (26%)	11 (31%)
Pain	30 (28%)	37 (35%)	21 (60%)*	5 (14%)
Rash	9 (8%)	18 (17%)	7 (20%)	7 (20%)

* = Significant difference at the level of *P* < 0.05. All other differences were not statistically significant

Among patients with metastatic cancer, 41% reported that the app helped them track their symptoms. Fifty-five percent of patients on adjuvant or neoadjuvant chemotherapy found the app helpful for symptom tracking, as did 57% of newly diagnosed patients.

### Clinical trial matching

At baseline, 47% of participants reported interest in information about clinical trials, and 54% reported awareness of clinical trial options while 34% did not. When asked at follow-up about their experience with the app, 35% reported that they were able to identify a clinical trial they were interested in learning about, including 50% of patients with a new diagnosis and 38% with metastatic disease. Overall, 33% of participants reported that they were more likely to consider a clinical trial after using the app.

### Patient experience and satisfaction with care

There was no significant change in patient distress or satisfaction with cancer care over the course of the study. At baseline, 56% of participants reported distress of 4 or greater on the NCCN distress thermometer, compared to 43% at the end of the study (*P* = 0.08). Patients generally reported high satisfaction with their patient/doctor relationship at baseline, with mean score on the PDRQ9 of 4.78, and it remained high at the end of the study period with mean score of 4.83.

**Fig. 3:**
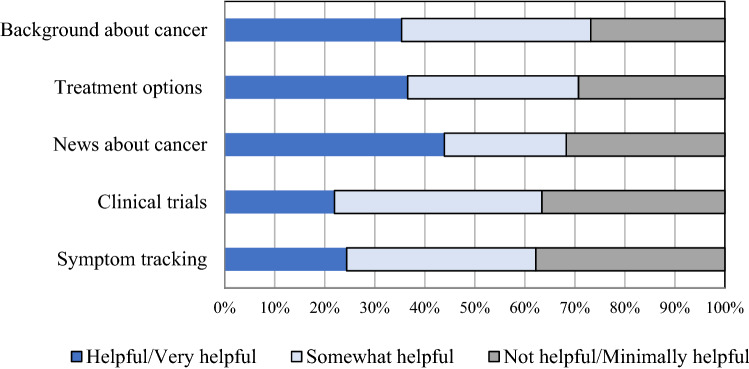
Participant Experience with Specific Features of the Outcomes4Me App. At the end of the 12 week study period, patients were asked which features of the app were most helpful. The top 5 categories from respondents (*N* = 82) are shown. Dark blue bars = Helpful/very helpful, light blue bars = somewhat helpful, gray bars = not helpful/minimally helpful

At baseline, we asked participants what healthcare information they were most interested in and the top areas of interest were: possible side effects of treatment (78%), prognosis (72%), and best treatment options (71%). Among the 82 participants who completed the end of study survey, the app features deemed most helpful were: Information about their specific type of breast cancer (76%), information about treatment options (74%), the personalized breast cancer newsfeed (70%), symptom tracking (65%), and clinical trial information (65%), as demonstrated in Fig. 3. Overall, 83% (68) of participants reported that the app was easy to use and 40% (33) reported that they plan to continue using the app following completion of the study.

## Discussion

There is a critical need to improve breast cancer patient engagement, education, and self-efficacy to allow for informed treatment decision making, effective symptom management and improved quality of life [1, 2, 4, 5]. An electronic tablet-based tool focused specifically on patient reported symptoms that are reported back to the treatment team has been shown to improve patient outcomes [9, 10]. However, despite considerable attention to these issues in the medical literature, there is no evidence-based tool that is consistently used to achieve all of these goals in routine practice. The Outcomes4Me app was developed as a patient navigation tool that could provide personalized information on diagnosis, treatment, clinical trial options, and symptom management. This app is available for free download by any patient with breast cancer but its impact on the patient experience has not been previously studied. We conducted a pilot study to evaluate the feasibility of integration of this app into the routine care of breast cancer patients and to explore features of the app deemed most helpful by patients and the characteristics of patients reporting greatest use and benefit from the app.

We found that inclusion of the Outcomes4Me app along with routine care for patients with any subtype or stage of breast cancer was feasible with 60% of patients engaging with the app at least 3 times over the 12-week study period. Engagement with the app was highly variable across the study population, with approximately 20% of participants engaging with the app 10 times of more, and close to one third of participants with minimal engagement. This seems consistent with preferences for use of a healthcare app in the general oncology population. The National Cancer Institute’s 2015 National Health Trends Survey found that 22% of respondents used a mobile health app, with greater usage associated with younger age, higher education, and higher income [19]. In our sample, higher education level was associated with increased app engagement, but age was not. Engagement was also higher among patients with newly diagnosed cancer compared to survivors and those living with metastatic cancer.

Similarly, the app was considered “useable” with an average SUS score of 70, above the standard threshold for average usability of 68 suggested in the literature [18]. Higher app engagement was associated with new cancer diagnosis and higher levels of patient education. While high SUS was seen across patients of all ages, and among those newly diagnosed and those on adjuvant chemotherapy, it was slightly below the usability goal for metastatic patients.

The population enrolled in this study was intentionally heterogeneous to allow for a preliminary assessment of how patients in different phases of breast cancer treatment interacted with the app. Increased app use in newly diagnosed participants was expected since these patients may be most interested in gathering information about their disease as part of planning their course of treatment. In addition, while the unmet needs including symptom management and treatment information among breast cancer survivors are well documented, it is not surprising that only a subset of patients in the survivorship phase of care had substantial engagement with the app during the study period. As prior research has demonstrated, survivorship needs among patients with breast cancer vary and at any given period in time, up to 40% of patients may have minimal needs [20]. Our study suggests the app may fulfill different needs for patients at different points in their breast cancer treatment.

One recognized challenge in the care of patients with breast cancer is the under-reporting of potentially sensitive topics including distress, cognitive problems, and sexual dysfunction in routine clinical care [21, 22]. While these symptoms were rare, we found that cognitive, sexual health and depression symptoms were more frequently reported by patients in the app than documented by providers in the EHR. These differences were not statistically significant and the study was not powered to evaluate significant differences in symptom reporting, thus this must be viewed as hypothesis generating for further study. Further, lack of documentation in the EHR does not mean these topics were not discussed, but the observed difference highlights the potential for app-based symptom assessment to identify quality of life issues that may not otherwise be reported. This is consistent with prior literature on the differences between prevalence of symptoms such as cognitive difficulty and sexual health problems and the degree to which they are discussed and addressed in clinic [23, 24]. Because under reporting may result in under treatment, improved identification of these issues may lead to more effective management. Future research could involve follow-up of such symptoms to evaluate whether they were discussed, how they were managed and whether this improved quality of life.

Many patients demonstrated interest in clinical trials at baseline. The majority of patients found the clinical trial information in the app helpful, over a quarter identified specific trials they wished to explore, and one third reported that the app experience made them more likely to consider clinical trials in the future. Because the majority of patients were already on treatment at the time of study enrollment and we do not know what trials were available to the newly diagnosed patients on the study, we were not able to evaluate the long-term impact of the Outcomes4Me app on trial enrollment. However, the number of patients reporting greater interest in trials is encouraging, given the historically low rate of clinical trial participation in adult oncology patients [25]. Future research will focus specifically on clinical trial matching with the app to better understand its potential role in facilitating clinical trial matching and enrollment.

There are several limitations to our study. First, we actively approached eligible patients to participate in the study and then provided assistance to download the app. Therefore, the patient population may not reflect the real-world spectrum of patients who would otherwise seek out and use such a navigation app. This would tend to bias results away from frequent engagement with the app. We did not prescreen patients for unmet information or navigation needs, or for specific interest in using an app to support their care, which might have boosted the level of engagement with the app during the study period. In addition, the study population was generally highly educated, recruited from a specialized breast cancer clinic, and at baseline reported high satisfaction with their provider, which may reflect a population at lower baseline need of supplemental information or navigation assistance compared to the general population of patients with breast cancer. In addition, we purposefully recruited patients across the full continuum of a breast cancer diagnosis, which allowed us to explore utility among subgroups of patients, but limited the number of patients within each subcategory. This was a single-arm study and we did not evaluate actual changes in care as a result of the intervention. This will be explored in future research. Another limitation was that only 75% of participants completed the final survey. Although there were no obvious differences in patient characteristics between the subsets that did or did not complete the end of treatment survey, it is possible that there may have been differences in the usability scores. The study feasibility assessment period was restricted to 12 weeks which is another limitation of the study and may have been too short to assess the feasibility.

In summary, we demonstrated that it is feasible to incorporate a patient navigation app into standard care for patients with breast cancer to provide information about treatment decisions, clinical trials, and symptom management. The app was generally deemed useful by patients as a means to learn more about their disease, identify and learn about clinical trials, and capture symptoms including some that may not be as well captured in clinic visits. Engagement with and perceived benefit from the app was highly variable, suggesting that this approach to education and navigation may be helpful for some, but not all patients with breast cancer, but usability and benefit was demonstrated across patients of all ages and education levels. In an unselected group of patients presenting for routine care, the Outcomes4Me app appeared particularly useful for patients with newly diagnosed cancer and for those on chemotherapy. With the ubiquity of mobile devices, this and other app-based approaches to patient education, symptom tracking and management and clinical trial matching hold promise to improve the care and outcomes for patients with breast cancer. Future research will explore how adaptation of the Outcomes4Me app to specific populations of patients with breast cancer can broaden its utility in all settings and evaluate its impact on care delivery.

## Supplementary Information

Below is the link to the electronic supplementary material.

Supplementary file1 (PDF 78 KB)

## Data Availability

The datasets generated during and/or analyzed during the current study are not publicly available to protect the privacy of individual study participants but are available from the corresponding author on reasonable request.
