# Supplementary material for: Feasibility of introducing a smartphone navigation application into the care of breast cancer patients (The FIONA Study)
Source: Breast Cancer Res Treat. 2023 Apr 27;199(3):501–9. doi: 10.1007/s10549-023-06918-y (PMC10132949; doi:10.1007/s10549-023-06918-y)
Supplement: Supplementary file 1 — Supplementary file1 (PDF 78 KB) [file 10549_2023_6918_MOESM1_ESM.pdf]

Feasibility of integrating a smartphone navigation application into the care of breast cancer patients (The FIONA Study)

Breast Cancer Research and Treatment

Steven J. Isakoff MD, PhD[1]; Maya R. Said ScD[2]; Agnes H. Kwak[1]; Eva Glieberman MS, MPH[2]; Emily A. O'Rourke[2]; Amanda Stroiney[2]; Laura Spring MD[1]; Beverly Moy MD, MPH[1]; Aditya Bardia MD, MPH[1]; Nora Horick MS[1]; Jeffrey M. Peppercorn MD, MPH[1]

[1]Massachusetts General Hospital Cancer Center, 55 Fruit Street, Boston, MA 02114, [2]Outcomes4Me Inc. 33 Arch Street, 17th Floor, Boston, MA 02110

Corresponding Author:

Steven J. Isakoff MD, PhD

Contact info: sisakoff@mgh.harvard.edu

Supplementary Table 1. Top Reported Symptoms in App and EHR by PRO-CTCAE Category include pain, gastrointestinal issues and sleep problems.

Category	N (%) who reported in app	N (%) who reported in EHR
Pain	30 (28%)	54 (50%)
Gastrointestinal	32 (30%)	48 (45%)
Sleep/Wake	31 (29%)	48 (45%)
Cutaneous	16 (15%)	33 (31%)
Neurological	18 (17%)	23 (21%)
Mood	19 (18%)	11 (10%)
Respiratory	8 (7%)	12 (11%)
Oral	10 (9%)	10 (9%)
Gynecologic/Urinary	7 (7%)	10 (9%)
Cardio/Circulatory	1 (1%)	12 (11%)
Attention/Memory	10 (9%)	2 (2%)
Visual/Perceptual	4 (4%)	4 (4%)
Sexual	5 (5%)	0 (0%)
Miscellaneous	18 (17%)	24 (22%)
Non PRO-CTCAE	2 (2%)	6 (6%)
